# Humanized TLR7/8 Expression Drives Proliferative Multisystemic Histiocytosis in C57BL/6 Mice

**DOI:** 10.1371/journal.pone.0107257

**Published:** 2014-09-17

**Authors:** Jessica M. Snyder, Piper M. Treuting, Lee Nagy, Cathy Yam, Jaehun Yi, Alicia Brasfield, Lisa Phuong Anh Nguyen, Adeline M. Hajjar

**Affiliations:** 1 Department of Comparative Medicine, University of Washington, Seattle, Washington, United States of America; 2 Comparative Pathology Program, University of Washington, Seattle, Washington, United States of America; University of Tokyo, Japan

## Abstract

A humanized *TLR7/TLR8* transgenic mouse line was engineered for studies using TLR7/8 ligands as vaccine adjuvants. The mice developed a spontaneous immune-mediated phenotype prior to six months of age characterized by runting, lethargy, blepharitis, and corneal ulceration. Histological examination revealed a marked, multisystemic histiocytic infiltrate that effaced normal architecture. The histological changes were distinct from those previously reported in mouse models of systemic lupus erythematosus. When the mice were crossed with *MyD88^−/−^* mice, which prevented toll-like receptor signaling, the inflammatory phenotype resolved. Illness may be caused by constitutive activation of human *TLR7* or *TLR8* in the bacterial artificial chromosome positive mice as increased TLR7 and TLR8 expression or activation has previously been implicated in autoimmune disease.

## Introduction

Toll like receptors (TLRs) are pattern recognition receptors important in the innate immune response, and 10 TLRs in humans and 12 TLRs in mice have been identified [1]. Mice and humans share TLR 1–9 in common, although there may be differential TLR function in mice and humans by ligand recognition and expression pattern [1], [2], [3]. For example, in the mouse, plasmacytoid dendritic cells (pDC) express both TLR7 and TLR8; whereas, in humans pDC express TLR7 and conventional or myeloid dendritic cells (cDC) express TLR8 [1], [4]. The various TLRs are stimulated by distinct bacterial, viral, or cellular components, and all TLRs signal through the myeloid differentiation primary response protein 88 (MyD88) dependent pathway except TLR3 [1]. TLRs 7 and 8 are intracellular and are expressed on the endosome membrane of B cells, pDC, and monocytes [1], [5]. Human TLRs 7 and 8 respond to viral single stranded (ss) RNA [5], [6], [7], and viral stimulation of TLR7 triggers production of type 1 interferons [8]. Mouse TLR8 differs from human TLR8 in not consistently recognizing ssRNA ligands or RNA viruses [4]. Stimulation of TLR7 by self-antigens such as endogenous RNA which enters the endosomal compartment as a result of necrosis or apoptosis of nearby cells has been well described [9], [10]. Increased expression and activation of TLR7 and TLR9 has previously been implicated in autoimmune diseases such as systemic lupus erythematosus (SLE) in BXSB and other lupus prone *Yaa* mice, which have a *Tlr7* duplication, and in humans [9], [11], [12], [13], [14], [15]. Humans with SLE have increased TLR7 expression in peripheral blood mononuclear cells which correlates significantly with IFNα mRNA [16]. *Yaa* mice have a mutation characterized by translocation of a portion of the X chromosome (which contains the gene encoding *Tlr7*) onto the Y chromosome, and male BXSB/*Yaa* mice develop severe autoimmune disease consistent with SLE, with approximately 90% of mice developing fatal immune glomerulonephritis by 8 to 9 months of age [15]. Male *Tlr7* ^−/−^/*Yaa* mice develop less severe lupus nephritis than *Tlr*7^+^/*Yaa* B6.*Nba2* congenic mice suggesting that increased copy number results in increased disease progression [12]. Recently, it has been reported that overexpression of mouse *Tlr7* in TLR7.1 transgenic mice causes an expansion of transitional 1 (T1) splenic B cells, resulting in the production of anti-RNA autoantibodies [17]. Similarly, deletion of mouse *Tlr8* was associated with autoimmunity and a reciprocal increase in the expression of *Tlr7* and decreased marginal zone B cells as well as peritoneal B1 B cells [18]. These mice showed increased production of autoantibodies and glomerulonephritis [18]. In addition, transgenic over-expression of human *TLR8* resulted in a wasting-disease and infertility requiring analysis of embryonic stem-cell chimeric mice in 3 of 4 lines tested [4]. These mice had severe pancreatitis with less pronounced pyelitis, glomerulonephritis, salivary adenitis, and periportal hepatitis. The chimeric mice that survived the longest developed spontaneous arthritis [4].

Previous reports have described “humanized” mice in which human tissues or cells are transplanted into immunodeficient mice to create rodent models which more closely mimic human cellular and humoral immune responses [4], [19], [20], [21]. Mice transgenic for human bacterial artificial chromosomes (BACs) encoding *TLR4* and its coreceptor MD-2 (which together form the LPS receptor complex) have also been crossed with mice containing targeted deletions of the mouse *Tlr4*/MD-2 to create a humanized *TLR4*/MD-2 mouse [21]. These mice display human-like recognition of LPS and are currently being tested in vaccine studies with TLR4 stimulating adjuvants. Here, we report a colony of humanized *TLR7/TLR8* (hu *TLR7/8*) mice that developed a severe immune mediated phenotype prior to 6 months of age, although the phenotype was distinct from previous reports of SLE-like disease in *Tlr*7^+^/*Yaa* congenic mice [9], [12]. The lesions were also distinct from the previously reported spontaneous lesions in humanized *TLR8* mice which consisted of a multisystemic mixed inflammatory cell infiltrate in the pancreas, liver, kidney, salivary glands, and joints [4]. The lesions in the currently described mice included proliferative multisystemic infiltrates of predominantly histiocytic cells admixed with variable numbers of lymphocytes and fewer neutrophils. Eyelids, exocrine pancreas, male and female reproductive tract, meninges, and subcutis were consistently involved, with near obliteration of normal architecture in severe cases. Significant glomerulonephritis was not present. In an attempt to ameliorate the phenotype and to determine whether disease was due to increased or abnormal signaling by the humanized TLR7 and TLR8 receptors, the mice were crossed with *MyD88* knockout mice, with resolution of the histiocytic inflammatory phenotype.

## Materials and Methods

### 1. Mice

**Figure 1:**
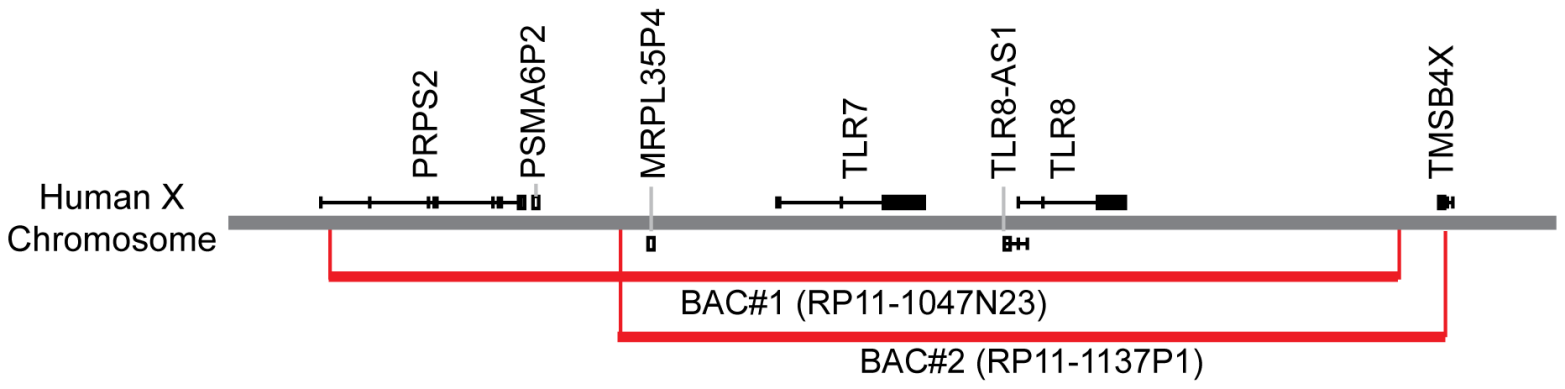
Schematic of human X-chromosome depicting location of BACs used to generate humanized TLR 7/8 mice. PRPS2  =  phosphoribosyl pyrophosphate synthetase 2; PSMA6P2  =  proteasome (prosome, macropain) subunit, alpha type, 6 pseudogene 2; MRPL35P4  =  mitochondrial ribosomal protein L35 pseudogene 4; TLR8-AS1  =  TLR8 antisense RNA 1; TMSB4X  =  thymosin beta 4, X-linked.

**Figure 2:**
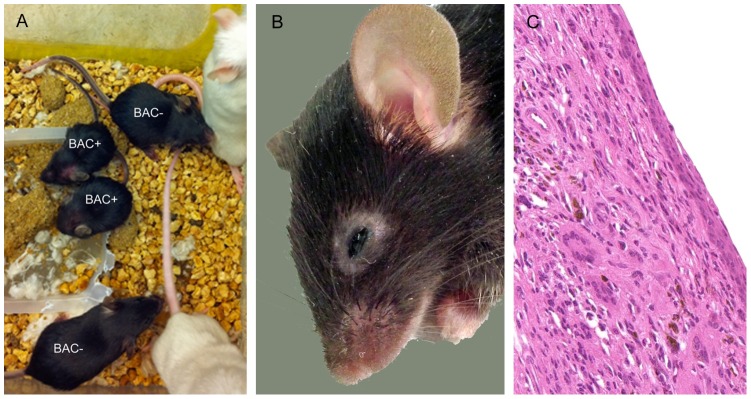
Gross and initial microscopic findings. A. BAC+ pups are noticeably smaller than BAC- littermates at 4 weeks of age. White mice are the recipient females of IVF eggs. B. With age, clinical signs included chronic blepharitis and periocular alopecia. C. Hematoxylin and eosin-stained section of affected eyelid in B. The submucosa is expanded by numerous histiocytes including multinucleated giant cells and melanophages, 20x.

Human genomic bacterial artificial chromosome (BAC) RP11-1047N23 (BAC#1) and RP11-1137P1 (BAC#2) were obtained from The BACPAC Resource Center (CHORI, Oakland, CA) (Figure 1). These were injected into fertilized C57BL/6N oocytes by the Transgenic Core at the University of Washington. Positive pups were identified by PCR (primer sequences shown in Table 1) and intercrossed with mice containing a targeted deletion of both mouse *Tlr7* and *Tlr8* (VelociGene Modified Allele ID #144) [22], obtained from Regeneron, to generate humanized *TLR7/8* mice (Table 2). *MyD88* knockout (KO) mice were obtained from Shizuo Akira (Osaka, Japan) and were backcrossed to C57BL/6J mice for ∼8 generations [23]. This study was carried out in strict accordance with the recommendations in the *Guide for the Care and Use of Laboratory Animals* of the National Institutes of Health. All protocols were approved by the Institutional Animal Care and Use Committee of the University of Washington. Mice were monitored by vivarium staff for general health daily and by the research group at least twice weekly. Recommendations of the veterinary staff were followed for mice reported for health concerns, including euthanasia of ill mice, usually by six months of age, to avoid pain and suffering. Humanized *TLR7/8* male mice were bred by *Tlr7/Tlr8* double knockout females. Occasionally, antibiotic ophthalmic ointment was used to treat corneal lesions that sometimes resulted from blepharitis (see Figure 2) in mice used as breeders.

**Table 1:** Polymerase chain reaction primer sequences used to identify positive pups.

Primer	sequence 5′ to 3′
CM-F	CGC TAC ACA GGC TCC TGA GA
CM-R	ACG GTC AGG ACC TGG ATT GG
5′BAC#1-F	ATTGTTGAGTCAGTGACGCTC
5′BAC#1-R	AAGATGTCTACATCCTAATCC
5′BAC#2-F	ACTGGGATTACAGGTGTGAGCC
5′BAC#2-R	ACTAACAGCAGATCTCTCCGC
3′BAC-F	ATCACGAGGTCAGGAGATTGAG
3′BAC-R	AATGCTCGATCACTGGCGTC
TLR7-F	AGCTTCCCAGAAAATGTCCTCAAC
TLR7-R	GTATGGTTAACCCACCAGACAAACC
muTLR8-F	AGTTGGATGTTAAGAGAGAAACAAACG
muTLR8-R	ATGGCACTGGTTCCAGAGGA
huTLR8-F	GACTCAGTTATTGCAGAGTGCAG
huTLR8-R	ATGATTCATTCGTTATGTGTGTGATGA
b-actin-F	TCCTTCGTTGCCGGTCCAC
b-actin-R	ACCAGCGCAGCGATATCGTC

**Table 2:** Number of injections, pups born, and lines generated for human genomic bacterial artificial chromosome (BAC) creation of humanized *TLR7/8* mice.

	Injections	Pups born	+ Lines
BAC #1	8	84	2
BAC #2	8	67	1

### 2. Cell culture

Bone-marrow derived macrophages (BMDM) were generated as previously described [21] with 1/3^rd^ of BM cells (∼2×10^7^ cells) plated per 15 cm dish. Dendritic cells (DC) were cultured in RPMI + glutamax + Na-pyruvate + non-essential amino acids (all from Life Technologies, Grand Island, NY) +0.05 mM BME and 10% (for Flt-3L-derived DC) or 5% (for GMCSF-DC) heat-inactivated fetal calf serum (Hyclone, ThermoFisherScientific, Waltham, MA). To generate Flt-3L derived BMDCs, 1/3 of BM cells were plated in 10 ml medium +100 ng/ml Flt-3L (Cedarlane Laboratories, Burlington, NC) in a 10 cm uncoated petri dish. On day 4, ½ of the medium was replaced with prewarmed medium containing Flt-3L. Cells were harvested on day 8 by pipetting up and down several times, spun, and resuspended in 3 ml medium without Flt-3L. GM-CSF derived BMDCs were generated by culturing ∼1×10^7^ BM cells in 10 ml medium containing 20 ng/ml GMCSF (ThermoFisherScientific) in 10 cm uncoated petri dishes. On day 2, 10 ml of prewarmed medium containing GMCSF was added to the plate, and on days 4 and 7, 10 ml was removed from the plate and replaced with fresh prewarmed medium containing GMCSF. Cells were harvested on day 10 by pipetting up and down several times, spun, and resuspended in 3 ml medium without GMCSF. Cells were washed with PBS prior to RNA extraction.

### 3. Splenocyte stimulation

Total splenocytes were stimulated as previously described [21] for a total of 5 hr in the presence of 10 µg/ml Brefeldin-A. R848, CL075, and Gardiquimod, all from InvivoGen (San Diego, CA) were added at 1 µg/ml and ODN1826, from Coley Pharmaceuticals (prior to acquisition by Pfizer, Dusseldorf, Germany) was added at 10 µg/ml.

### 4. Flow cytometry

Spleens were prepared and stained as described [21] using the following surface antibodies for cell enumeration: biotinylated Siglec H (Hycult Biotech) and streptavidin-APC (BD Biosciences), CD11c PE-Cy7 (BD Biosciences), CD11b PerCP-Cy5.5 (Biolegend), CD19 APC-Cy7 (BD Biosciences), CD3e Pacific Blue (Biolegend), Ter-119 FITC (Biolegend). For intracellular cytokine staining the following antibodies were used: biotinylated Siglec H (Streptavidin-APC), CD11c PE-Cy7, CD11b PerCP-Cy5.5, CD19 APC-Cy7, TNF Alexa 700 (BD Biosciences), IL-12p40 PE (BD Biosciences), IFNalpha FITC (Antigenix).

### 5. Quantitative real time PCR

Relative TLR7 and TLR8 expression levels were determined by real-time PCR using an ABI 7300 Real Time PCR System and the Brilliant II SYBR Green QPCR reagents (Agilent Technologies Inc., Santa Clara, CA). Total RNA was isolated from cells or tissues using Trizol, DNase treated, and reverse transcribed using oligo dT priming and Superscript II (Invitrogen, Carlsbad, CA). β-actin was used to normalize the data between samples. Table 1 lists the primers used.

### 6. Intraperitoneal (IP) injections and serum cytokine analysis

Mice were injected with 100 µg R848 (Resiquimod; InvivoGen, San Diego, CA) in 0.2 ml PBS IP. Mice were then bled from the retroorbital sinus 1 hr after injection, and a terminal cardiac puncture was obtained at 3 hr after injection. Cytokines were measured in filtered serum diluted 1∶3 in sample diluent using a Bio-Plex Pro Assay kit according to manufacturer instructions, and ran on a Bio-Rad Bio-Plex 200 system.

### 7. Histopathology

**Figure 3:**
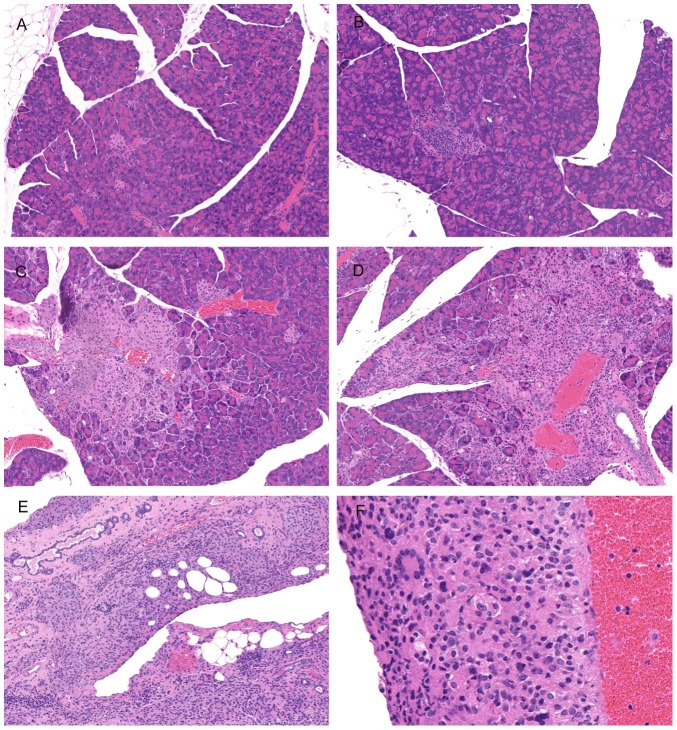
Representative hematoxylin and eosin-stained examples of pancreatic inflammation scores. A. Grade 0: Normal pancreas, 10x; B. Grade 1: Rare clusters of histiocytes with preservation of normal architecture, 10x; C. Grade 2: Coalescing clusters of histiocytic cells, 10x; D. Grade 3: Significant histiocytic infiltrate with destruction of normal tissue architecture, 10x; E. Grade 4: Near obliteration of normal tissue architecture by histiocytic infiltrate, 10x; F. Significant histiocytic infiltrate disrupting normal tissue architecture with presence of multinucleated giant cells, 20x.

Complete gross examination was performed following euthanasia via carbon dioxide asphyxiation in 54 animals ranging from 1 month to 7 months of age. Tissues from 47 mice were fixed with 10% neutral buffered formalin and routinely embedded in paraffin, and stained with hematoxylin and eosin (H&E), and kidneys from 7 mice (5 BAC+, 1 BAC- control and one unrelated C57BL/6 control mouse) were frozen in Tissue-Tek O.C.T. compound (Sakura Finetek U.S.A., Torrance, CA) for IgG immunohistochemistry. 27 mice initially presented were subjected to complete necropsy and all major organs (decalcified cross section head, skin, lung, heart, liver, kidney, adrenals, spleen, pancreas, lymph nodes, salivary gland, gastrointestinal tract, reproductive tract) were examined histologically. Follow up morphologic phenotyping limited the histology analysis to the target tissues and included decalcified cross sections of the head, pancreas, mesenteric lymph nodes, and reproductive tissues. Lesions were graded on a 0 to 4 scale based on their severity and extent, with 0 representing normal tissue, 1 representing presence of low numbers of histiocytic cells with preservation of normal architecture, 2 representing coalescing histiocytic inflammatory infiltrates, 3 representing significant histiocytic infiltrate with destruction of normal tissue architecture, and 4 representing near obliteration of normal tissue architecture. Lesions were also graded for the presence of multinucleated giant cells (Figure 3, A–F).

### 8. Immunohistochemistry

IgG immunohistochemistry (IHC) was performed on O.C.T. frozen tissues. Briefly, tissues were primed with PBS and antigenic sites were blocked with 10% normal goat serum in TBS for 30 minutes. Primary antibody (AF 488 goat anti-Mouse IgG (H+L); Life Technologies, Grand Island, NY) at a 1∶200 dilution was incubated for 1 hour followed by 3 washes with PBS and staining with 1∶1000 DAPI. Slides were then mounted with mounting medium and coverslipped. Negative controls were included in which PBS was substituted for primary antibody. All staining procedures were performed by hand. For CD3, CD45, MAC2, and F4/80 IHC, slides were deparaffinized on the Leica Bond Automated Immunostainer (Leica Biosystems, Buffalo Grove, IL). For CD3, CD45, and MAC2 IHC, antigenic sites were exposed by heating in a citrate buffer (Bond HIER1; Leica Biosystems, Buffalo Grove, IL) for 10 minutes at 100°C followed by blocking in a 10% Normal Goat Serum in PBS. All staining procedures were run on the Leica Bond Automated Immunostainer. Primary antibodies rat anti-mouse CD45R (BD Biosciences, San Jose, CA) at a 1∶5000 (0.1 µg/mL) dilution, rat anti-human CD3 (Bio-Rad, Raleigh, NC) at a 1∶500 (2 µg/mL) dilution, and rat anti-mouse MAC2 (Cedarlane, Burlington, Ontario) at a 1∶10,000 (0.1 mg/mL) were incubated for 30 min at room temperature (RT) followed by secondary antibody (rabbit anti-rat 1∶300+5% Normal Goat Serum in TBS; Vector Labs, Burlingame, CA) incubated for 8 min at RT. Slides were then treated with Leica Polymer (Leica Biosystems, Buffalo Grove, IL) for 8 minutes at RT, Bond Peroxidase blocker (Leica Biosystems, Buffalo Grove, IL) for 10 minutes at RT, and Leica Refine (DAB) detection kit (Leica Biosystems, Buffalo Grove, IL) for 10 minutes. Slides were counterstained with hematoxylin, cleared to xylene, and mounted with synthetic resin mounting medium. For F4/80 IHC, antigenic sites were exposed using Bond Enzyme pre-treatment (Proteinase K; Leica Biosystems, Buffalo Grove, IL) for 10 min at 37°C followed by blocking in 10% Normal Goat Serum in TBS. Primary antibody (rat anti-mouse F4/80 CALTAG; Life Technologies, Grand Island, NY) at a dilution of 1∶200 (1 µg/mL) was incubated for 30 minutes at RT followed by secondary antibody (rabbit anti-rat 1∶300+5% Normal Goat Serum in TBS; Vector Labs, Burlingame, CA) incubated for 8 min at RT. Slides were then treated with Leica Polymer (Leica Biosystems, Buffalo Grove, IL) for 8 minutes at RT, Bond Peroxidase blocker (Leica Biosystems, Buffalo Grove, IL) for 10 minutes at RT, and Leica Refine (DAB) detection kit (Leica Biosystems, Buffalo Grove, IL) for 10 minutes. Slides were counterstained with hematoxylin, cleared to xylene, and mounted with synthetic resin mounting medium.

### 9. Statistical analysis

Statistics were performed in GraphPad Prism 5 (San Diego, California). Correlation of histologic severity score over time was tested using least squares fit method and R^2^ value was calculated using goodness of fit. The two-tailed Mann Whitney test was used to test for differences between males and females. Comparisons of multiple groups were tested using the Kruskal Wallis test and the Dunn's Multiple Comparison's Test. A *P*-value of <0.05 was considered statistically significant.

## Results

### Generation of hu*TLR7/8* BAC+ mice

A genomic BAC transgenic approach was used to direct tissue-specific expression of human *TLR7* and *TLR8* to generate a mouse model for studies using TLR7/8 ligands as vaccine adjuvants. Mice transgenic for human BACs encoding *TLR7* and *TLR8* were intercrossed with mice containing targeted deletions of both mouse *Tlr7* and *Tlr8*, thereby replacing the mouse receptors with the human receptors. Two independent hu*TLR7/8* BAC+ lines were generated from BAC#1 (Figure 1, Table 2). The first line produced from BAC#1, a female, was euthanized at 3 months for respiratory distress, prior to breeding. The second line produced from BAC#1, a male, was euthanized at 2 months of age, prior to breeding, for penile prolapse. Sperm was obtained from this male before euthanasia, and *in vitro* fertilization (IVF) was performed. Two pups born as a result of IVF were runted and lethargic compared to wild type littermates and were euthanized at 4 weeks (Figure 2A, Table 3).

**Table 3:** Clinical features and outcome of mice from BAC#1 and BAC#2.

Mice	BAC	Sex	Age euthanized	Presentation
Line 1 founder	BAC #1	F	3 mo	Respiratory distress
Line 2 founder	BAC #1	M	2 mo	Penile prolapse
IVF pups Line 2	BAC #1	M+F	4 wk	Runts
Line 3 founder	BAC #2	M	5 mo	Hunched

Because BAC#1 encoded most of phosphoribosyl pyrophosphate synthetase 2 (PRPS2), a gene identified as a myc-target [24], a second BAC that lacked all PRPS2 sequences was subsequently used to produce a third line. Unlike the BAC #1 lines, these mice survived long enough to reproduce; however, BAC+ mice routinely developed clinical signs including blepharitis (Figure 2B–C), corneal ulceration, hunching, and loss of condition which required euthanasia by 4 to 7 months of age.

### Pathological observations in hu*TLR7/8* BAC+ mice

All 4 mice from BAC #1 (including the IVF pups) had postmortem examinations which showed mucometra in the mouse from the first line and lymphadenopathy and hepatosplenomegaly in two mice from the second line. Histopathological abnormalities included variably severe histiocytic inflammation with infiltration of the meninges, eyelids, pancreas, and reproductive tract in all cases and salivary gland, liver, kidney, skin, and subcutis in most cases (Table 4). The histiocytic infiltrate effaced some tissues while sparing other structures, most notably the islets of the pancreas (Figure 3, Figure S1A and S1B). Abnormalities on gross examination of BAC#2 mice included blepharitis and corneal ulceration, mild uterine enlargement, mild mesenteric lymphadenopathy, and enlargement of the epididymis. Histopathological abnormalities in these mice were often less pronounced than those of the BAC #1 lines but consistently included variably severe histiocytic inflammation and infiltration in the eyelids, meninges, pancreas, and reproductive tract, often with multinucleated giant cells present (Figure 2, Figure 3). Many cases had histiocytic infiltration of the subcutis, skin, and salivary gland; however, involvement of the liver and kidney was less consistent (Table 4).

**Table 4:** Clinical and pathological features of mice by genotype.

Genotype	Number (M/F)	Median age - days (range)	Clinical Signs	Median Meninges Score	Median Eyelid Score	Median Pancreas Score	Median Repro Score M/F	Other
No BAC Littermates	9 (5/4)	35 (18–210)	None	0	0	0	0/0	Mild to minimal periportal hepatitis (3/9); mild glomerulonephritis (1/7); preputial adenitis (1)
BAC#1+	4 (2/2)	45 (28–90)	Penile prolapse (1); respiratory distress (1); runted (2); hepato-splenomegaly (2); lymphadenomegaly (2)	3	2.5	4	2.5/4	**Salivary gland** (3/4); **SQ/dermis** (4/4); mild to severe interstitial nephritis (3/4); mild to moderate hepatitis (4/4); moderate pneumonitis (3/4)
BAC#2+ *MyD88* WT	23 (14/9)	114.5 (18–210)	Blepharitis and/or corneal ulceration (13); penile prolapse (1); splenomegaly (6); lymphadenomegaly (6)	2	3.5	3	3.5/3	**Salivary gland** (9/20); **SQ/dermis** (8/20); mild to moderate hepatitis (17/17); mild glomerulonephritis or interstitial nephritis (7/15); mild pneumonitis and perivasculitis (2/5)
BAC#2+ *MyD88* KO	7 (1/6)	90 (70–166)	Abscess (1); pregnant (1)	0	0	0*	0/0	*mild lymphocytic infiltrates in pancreas (4/6); liver extramedullary hematopoiesis (4/5); mild periportal hepatitis (2/5); lymphocytic aggregates in kidney (2/4)
BAC#2+ *MyD88* het	6 (6/0)	104.5 (90–158)	Blepharitis (4)	1	1	2	3/na	**Salivary** (1/4); **SQ/dermis** (2/5); periportal hepatitis (2/2); mild glomerulonephritis or interstitial nephritis (3/3)

Repro =  female or male reproductive organs; SQ =  subcutaneous; WT =  wild type; K0 =  knockout; het  =  heterozygote.

Lesions of the kidney in the second BAC #1 line consisted of histiocytic inflammation involving the pole of the kidney and capsule in all three cases and variably severe multifocal to coalescing histiocytic and lesser lymphocytic and neutrophilic interstitial nephritis in two cases (Figure S2A). The mouse from the first BAC #1 line did not have significant histologic renal changes. Mice from both BAC #1 lines had mild to moderate histiocytic and lesser lymphocytic centrilobular and occasionally periportal hepatitis in all four cases (Figure S2B). Mice from the BAC #2 line had much less dramatic histological hepatic and renal lesions, with mild periportal and centrilobular histiocytic and lymphocytic infiltrates and extramedullary hematopoiesis observed in the liver in most cases and minimal to mild membranoproliferative glomerulonephritis observed in the kidneys in approximately 50% of cases (Figure S1C–D and S1E–F). Histologically, the spleens from the BAC #2 line were enlarged secondary to moderate diffuse extramedullary hematopoeisis with all lineages represented (Figure S1G and S1H). Histological examination of spleens from the BAC #1 and infrequently the BAC#2 lines showed extension of histiocytic cells along the capsule of the spleen similar to the histiocytic proliferation extending from the renal capsule. Lymph nodes from mice of both lines showed reactive changes (follicle formation with germinal centers and sinus and medullary histiocytosis and plasmacytosis) but no pathologic histiocytic proliferation similar to that observed in target organs (Figure S1I and S1J). Bone marrow in decalcified cross sections of the skull was variably hypercellular with increased myeloid and lesser erythroid precursors, but also showed no obvious histiocytic proliferation.

**Figure 4:**
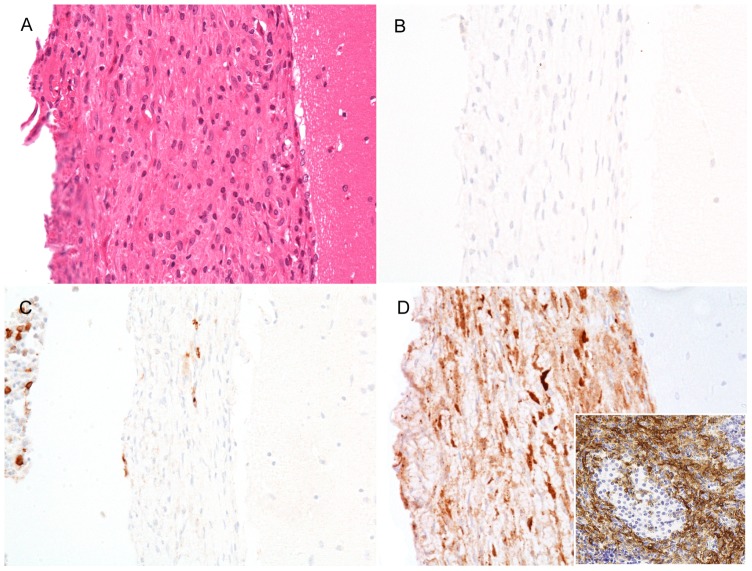
Inflammatory infiltrates are composed of MAC-2 expressing histiocytes. A. H&E-stained section of meninges for orientation of serial sections (B–D). B. Infiltrating cells are negative for CD3 (T-cells). C. Few cells within the thickened meninges are positive (brown) for the B cell marked CD45R (B220). Note bone marrow contains few B-cells (left side of image). D. Majority of infiltrating cells are MAC-2 positive histiocytes. Inset: Pancreas immunostained for the macrophage marker, F4/80. Note that the islet does not stain. All images 20x.

Immunohistochemistry for CD45, CD3, and MAC2 was performed in 2 male mice, one from each BAC. F4/80 IHC was performed on 2 mice from BAC#1 and one mouse from BAC#2. Atypical infiltrating cells in the meninges, pancreas, and reproductive organs were negative for CD45 and CD3 reactivity. MAC2 and F4/80 IHC showed strong positivity of infiltrating atypical cells in the meninges and pancreas (Figure 4). IgG IHC showed mild to moderate positivity in the glomeruli of 6 BAC#2 male mice (data not shown); however, intensity and extent of staining subjectively did not differ in BAC+ mice and the BAC- and C57BL/6 control mice.

### Correlation of lesion severity with age and sex

**Figure 5:**
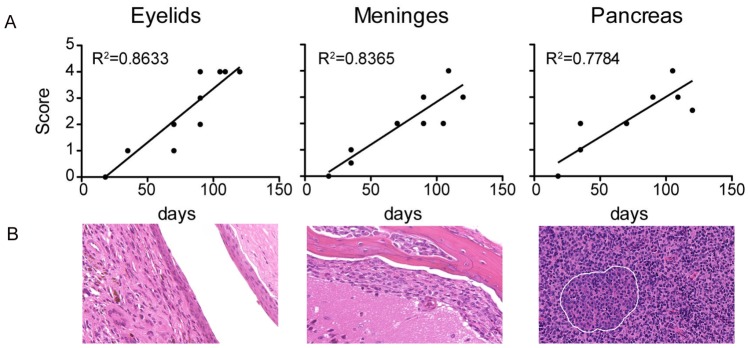
Severity of histiocytic inflammation increases with age. Histologic lesions were scored 0–4 and correlation was tested in BAC#2+/*MyD88*WT mice up to 120 d. Thirteen mice are analyzed. A. In eyelids, meninges and pancreas, histiocytic infiltrates increases in severity with age. B. Hematoxylin and eosin-stained representative examples of severe inflammation in tissues as indicated (20x). White line demarcates a pancreatic islet.

Lesions were identified in humanized *TLR 7/8* mice as early as 3 weeks of age (BAC #1) and 14 weeks of age (BAC #2) grossly. Mice from the BAC #2 line were examined histologically at 18 days of age, and no consistent lesions were identified. At 5 weeks of age, however, mild lesions were observed histologically, and by 10 weeks of age lesions were more robust. There was a strong correlation between age and lesion score over the first 120 days of age for pancreas (R^2^ = 0.7784), eyelid (R^2^ = 0.8633) and meninges (R^2^ = 0.8635) (Figure 5). Histologic changes were analyzed by gender using a two tailed Mann-Whitney test. The median age of female and male mice examined histologically was not significantly different (*P* = 0.35). Lesion severity scores for the meninges, eyelids, and reproductive organs were not statistically different in males and females; however, the median lesion severity score for the pancreas was significantly higher in males than in females (median lesion severity score 4 versus 2.75; *P* = 0.03).

### Ancillary diagnostic tests and flow cytometry

Complete blood counts were performed in 5 BAC+ mice, 1 from the second BAC #1 line and 4 from the BAC#2 line, and in 4 BAC- mice, and all were within normal limits. Serum chemistry was performed in one case from the BAC#2 line, and was unremarkable. One mouse with bilateral corneal ulceration had *Pasteurella pneumotropica* cultured from the eye, and in one mouse cultures of ocular discharge and uterine contents resulted in no aerobic growth. Because we were not able to maintain the lines generated from BAC#1 due to the severity and early lethality of the lesions, all the remaining analyses are from the BAC#2 line.

**Figure 6:**
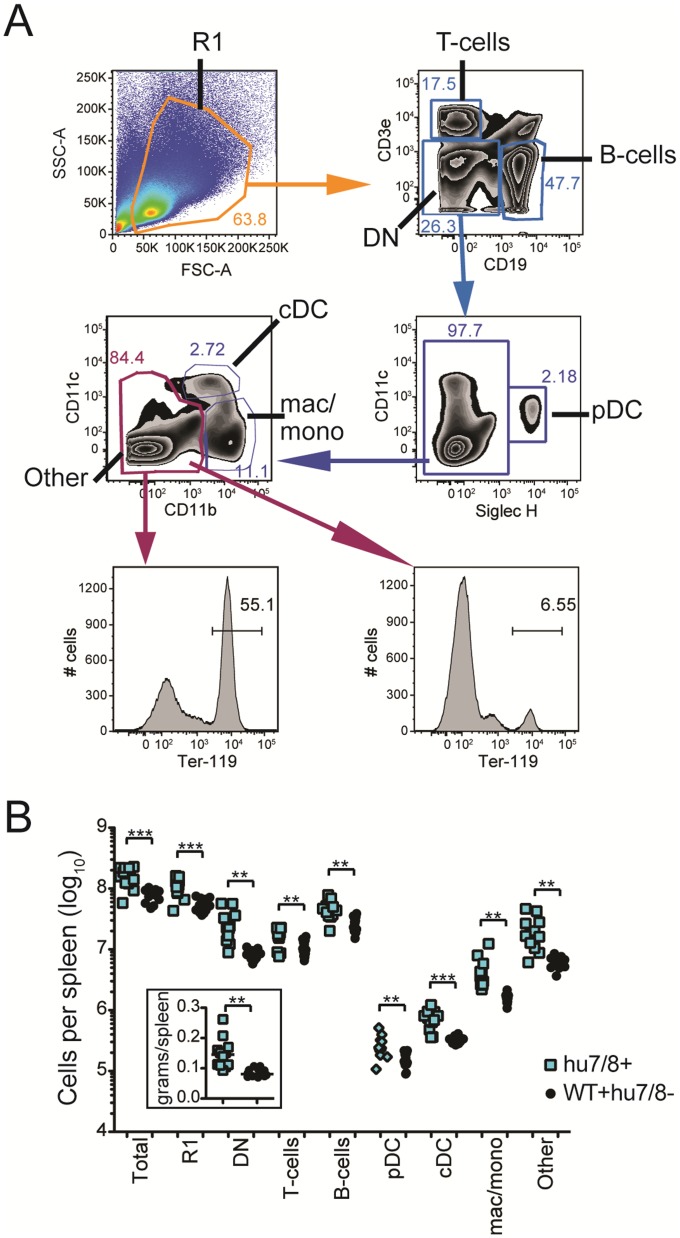
Fluorescent activated cell sorting analysis of spleens from hu*TLR7/8* BAC+ mice and WT controls. A. Gating strategy to identify the various cell populations in the spleen. B. Total cells per population per spleen were calculated based on FACS results and total cell counts. Inset shows weight of spleen per mouse. N = 12 per group. **P<0.01, ***P<0.001.

Fluorescent activated cell sorting (FACS) analysis of spleens from hu*TLR7/8* BAC+ mice and their wild type controls showed that all of the cell types tested (T-cells, B-cells, pDCs, cDCs, and mac/monos) were significantly increased in number in the hu*TLR7/8* BAC+ mice versus the wild type control mice (Figure 6), as shown for mu*Tlr7* BAC+ mice [25]. Occasionally, the “Other” population was expanded, but further staining with Ter-119 demonstrated that these expanded cells were due to extramedullary hematopoiesis as previously shown for mice transgenic for mu*Tlr7* [9]. Overexpression of mouse *Tlr7* results in markedly enlarged spleens (>0.3 g) [9], [25], however the spleens of hu*TLR7/8* BAC+ mice, though showing significantly higher median weights and cell numbers compared to littermate BAC- and C57BL/6 controls, were <0.3 g (Figure 6B, inset).

### Analysis of function of human *TLR7* and *TLR8* in hu*TLR7/8* BAC+ mice

**Figure 7:**
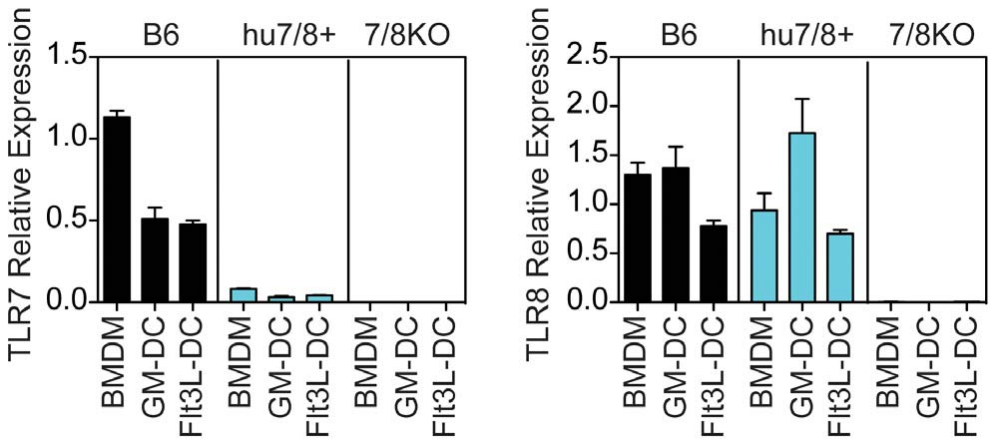
Alteration of TLR7 and TLR8 expression in hu*TLR7/8* BAC+ mice. Total RNA was extracted from BMDM, GMCSF and Flt-3L-derived DC and subjected to real-time PCR analysis. BMDM and Flt3L-derived DC results represent the average of three separate cell preparations and GMDC results represent the average of two separate cell preparations. The same primers with equivalent amplification efficiency were used for muTlr7 and huTLR7. However, separate primers had to be designed for muTlr8 and huTLR8 and thus the relative abundance of TLR8 cannot be directly compared between B6 and hu*TLR7/8* BAC+ mice. Data are presented as mean and range (GMDC) or standard error of the mean (BMDB, Flt3LDC).

We next sought to determine whether transgenic expression of human *TLR7* and *TLR8* restored responsiveness to TLR7/8 ligands in a *Tlr7*/*Tlr8* DKO background. We first determined RNA expression in bone-marrow derived macrophages (BMDM), GMCSF-derived dendritic cells (GMDC) and Flt-3Ligand-derived DC (Flt-3L DC; Figure 7). *Tlr7/Tlr8* double knockout mice were confirmed to lack expression of TLR7 and TLR8 in all three cell types. Although there was relatively less expression of *TLR7* by humanized mouse than *Tlr7* by C57BL/6 mouse cells, expression of hu*TLR7* and *huTLR8* in the various huTLR7/8 mouse cell types was confirmed. The pattern of expression by huTLR7/8 mouse cells also differed slightly, with a proportionally greater expression of TLR7 by Flt-3Ligand-derived DC compared to BMDM in huTLR7/8 cells compared to C57BL/6 cells (TLR7 expression in Flt-3L-derived DC was 0.4-fold that in BMDM in C57BL/6 cells compared to 0.6 fold in humanized TLR7/8 cells suggesting a more human-like expression pattern) (Figure 7). Increased expression of TLR8 was observed in BMDM compared to Flt-3L DC for both C57BL/6 and humanized mouse cells with greatest relative expression in GM-DC compared to other cells types in humanized TLR7/8 cells (Figure 7).

**Figure 8:**
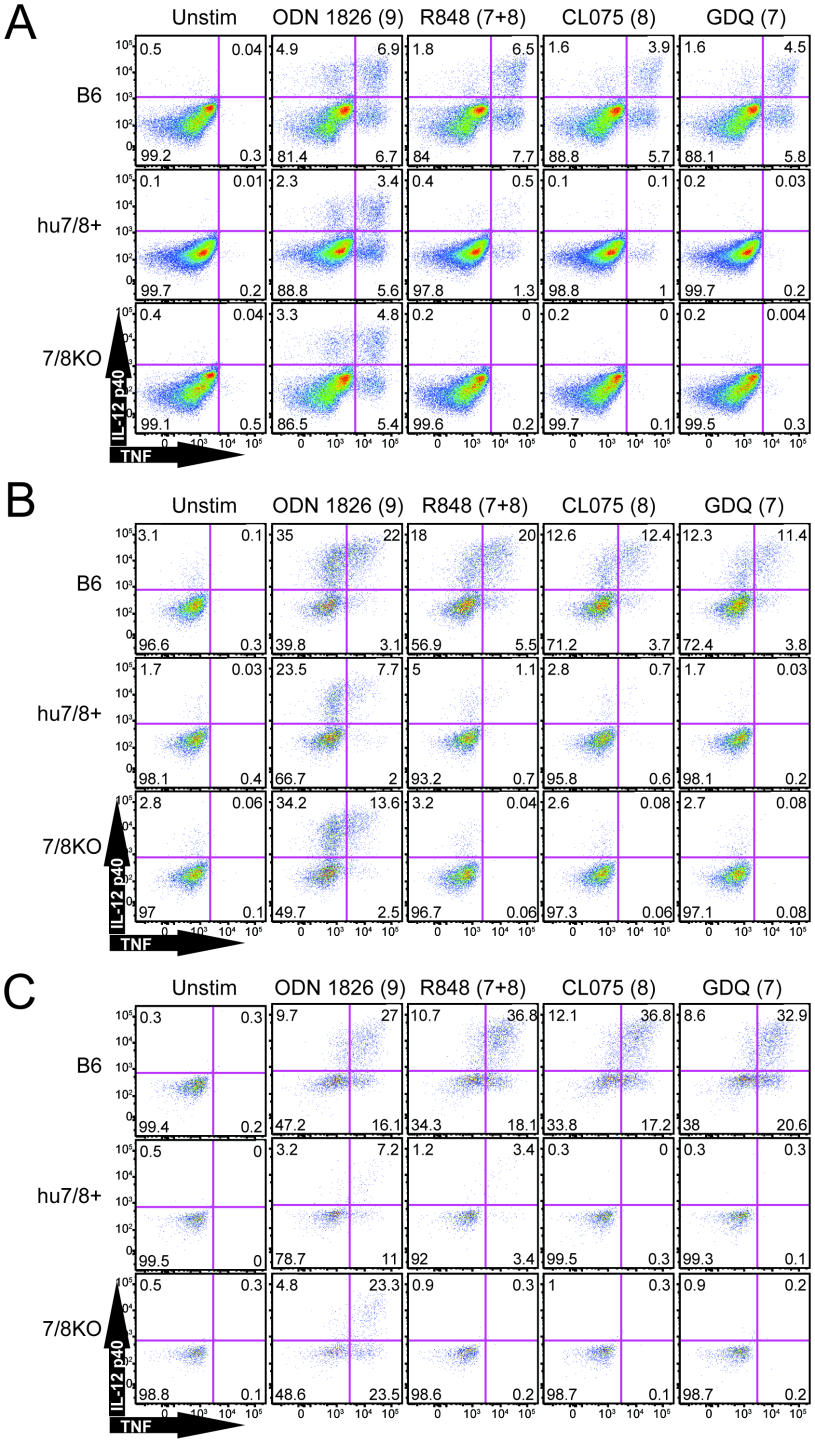
Intracellular cytokine induction in response to TLR7 or TLR8 ligands mirrors RNA expression. Whole splenocytes were stimulated directly *ex vivo* with the indicated ligands (the numbers in parentheses indicates the respective TLR that mediates the response to that ligand. Intracellular TNF (x-axis) and IL-12p40 (y-axis) production was examined in A) monocyte/macrophage, B) cDC, and C) pDC populations. The % of cells found in each quadrant is indicated in each corner. Shown is 1 of 2 experiments with similar results.

We next chose to confirm protein expression by examining functional responses. We stimulated whole splenocytes *ex vivo* and examined intracellular cytokine production in response to TLR7/8 ligands (Figure 8). The greatest response was observed in the macrophage/monocyte population in response to R848 (resiquimod), which stimulates both TLR7 and TLR8, although the % of cells responding in the humanized TLR7/8 cells was greatly reduced compared to C57BL/6 cells (Figure 8A). As predicted from the expression pattern in humans, the humanized splenic macrophage/monocyte population responded to a TLR8 ligand but not a TLR7 ligand. This is in contrast to the mouse expression pattern, where both receptors are expressed in macrophages and the macrophage/monocyte population responded to both ligands. CL075 that stimulates TLR8 preferentially, but not guardiquimod (GDQ) that stimulates TLR7 preferentially, stimulated the humanized TLR7/8 macrophage/monocyte population (and to a lesser extent cDC) even though both compounds stimulated B6 cells to an equivalent extent (Figure 8A and Figure 8B). Only R848, however, and not GDQ, stimulated hu*TLR7/8* BAC+ splenic pDC (Figure 8C), despite expression of human TLR7 RNA in bone-marrow derived pDC (Figure 7).

**Figure 9:**
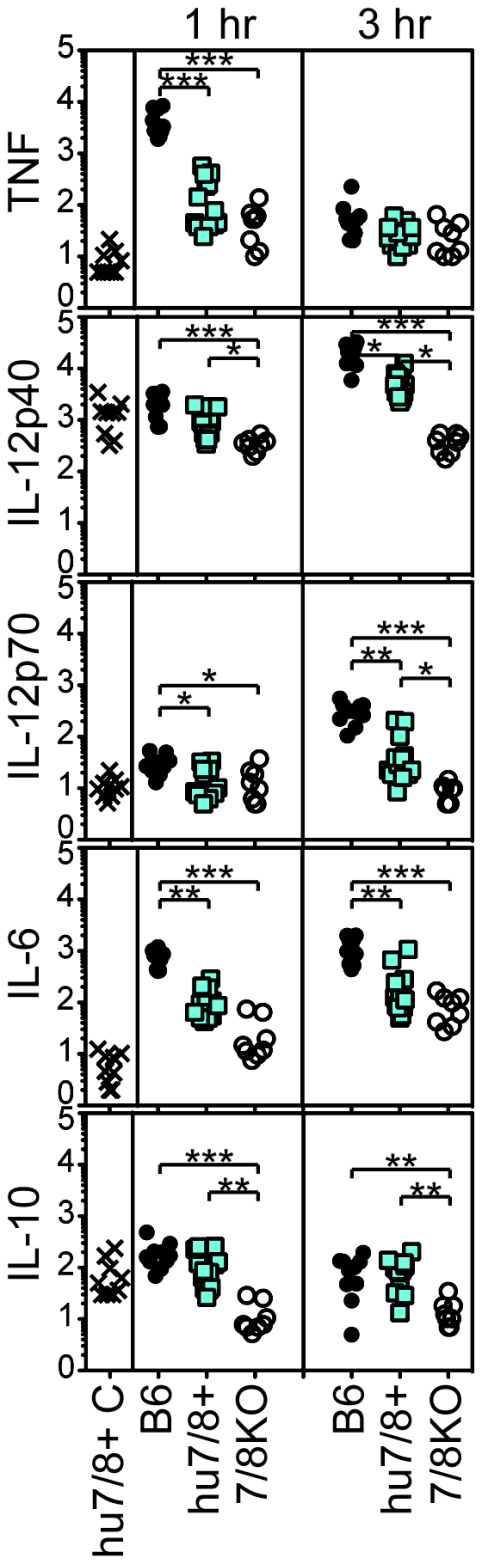
Poor *in vivo* cytokine induction in response to R848 in hu*TLR7/8* BAC+ mice. Each symbol represents one mouse and the same mice were bled at 1 and 3 hr post-injection. Shown are combined data from 3 separate experiments. N = 10 B6, 13 hu*7/8* BAC+, 8 7/8 DKO and 9 control (C) uninjected hu *TLR7/8* BAC+ mice. *P<0.05, **P<0.01, ***P<0.001.

Finally, to determine whether these mice might be useful for our vaccine studies, we measured serum cytokine production 1 and 3 hr following systemic administration of R848 (Figure 9). We also measured serum cytokines in control uninjected hu*TLR7/8* BAC+ mice (labeled hu7/8+ C in Figure 9). Wild-type C57BL/6 mice clearly induced TNF, IL-12 p40 and p70, IL-6, and IL-10 cytokines in response to R848 to levels greater than those induced in hu*TLR7/8* BAC+ mice. Even though IL-12p40 and IL-10 were significantly increased in hu*TLR7/8* BAC+ mice compared to *Tlr7/8* DKO mice, the control uninjected hu*TLR7/8* BAC+ mice also showed a trend towards higher levels than the DKO mice suggesting that human TLR7 or TLR8 might be constitutively activated in the BAC+ mice.

### Effect of crossing hu*TLR7/8* mice with *MyD88* ^−/−^ mice

**Figure 10:**
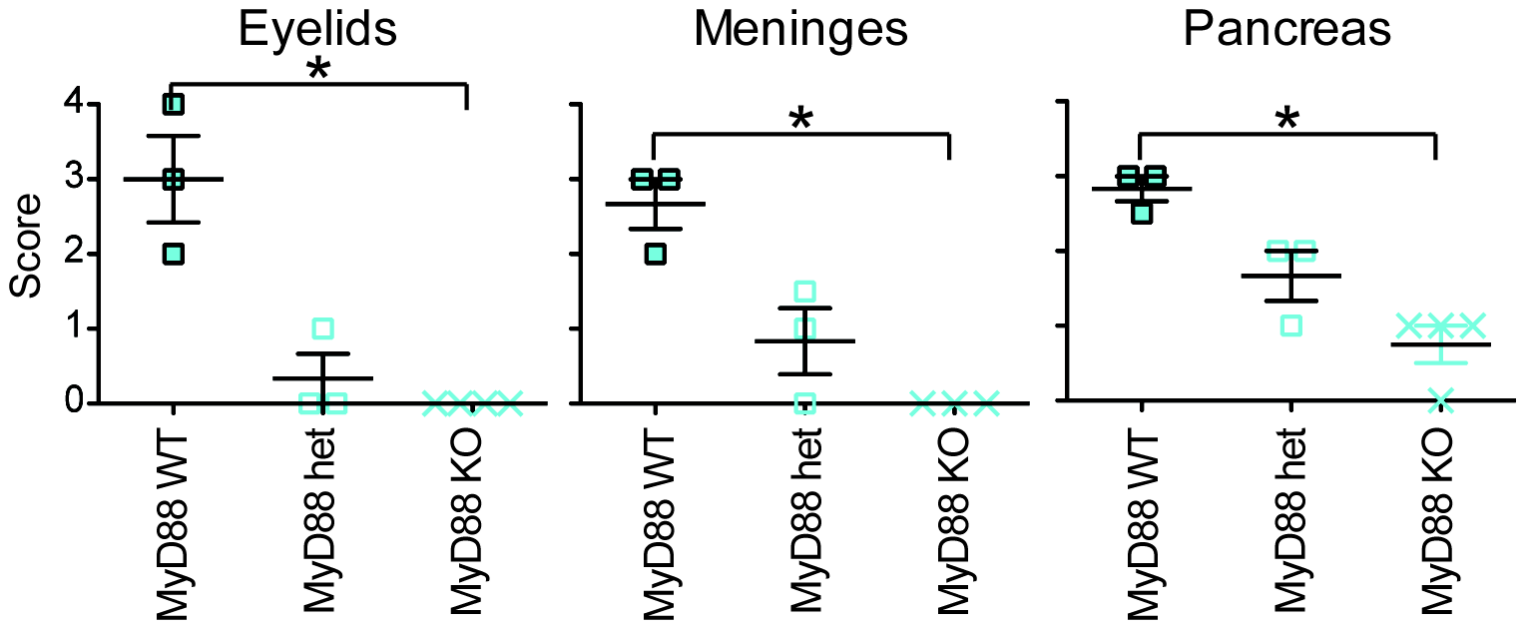
Histologic lesion severity score by genotype in three month old mice. For each organ, the severity score is highest in the hu7/8 BAC+ x *MyD88* WT mice (left group), intermediate in the hu7/8 BAC+ x *MyD88* heterozygote mice (middle group), and lowest in the hu7/8 BAC+ x *MyD88* knockout mice (right group). In the hu 7/8 BAC+ x *MyD88* KO mice, histology of the eyelids and the meninges is normal at three months (severity score 0). In the pancreas, there are occasional clusters of inflammatory cells at three months of age in the hu 7/8 BAC+ x *MyD88* KO mice; however the inflammatory cell infiltrate in these mice is composed predominantly of lymphocytes with rare histiocytes, rather than the typical histiocytic infiltrate seen in BAC+ mice. An asterisk shows groups that are significantly different (p<0.05).

In an attempt to rescue the phenotype, the hu*TLR7/8* BAC+ mice were crossed with *MyD88* knockout mice to generate hu*TLR7/8* BAC+ on *MyD88* ^−/−^ mice. The resultant mice did not display the previously demonstrated clinical signs of blepharitis and corneal ulceration, and mice were euthanized between 10 and 24 weeks of age to analyze pathological changes. Gross abnormalities included submandibular abscessation in one mouse, which was considered consistent with immunosuppression secondary to the *MyD88* ^−/−^phenotype [26]. The *huTLR7/8* BAC+ on *MyD88^−^* ^/−^ mice did not have significant histiocytic infiltration or inflammation in any organ, although tertiary lymphoid aggregates were identified in multiple tissues and considered consistent with the *MyD88* ^−/−^ phenotype. Age matched littermate hu*TLR7/8* BAC+ *MyD88* heterozygote and *MyD88* wild type controls were examined, and histiocytic inflammatory lesions similar to those previously identified in the original BAC#1 and BAC#2 mice were seen (Table 4). The median lesion severity score at 3 months of age for the BAC+ *MyD88* wild type mice was significantly higher than for the BAC+ *MyD88* knockout mice for meninges (p = 0.03), eyelids (p = 0.02), and pancreas (p = 0.02) (Figure 10). Lesion severity scores were intermediate for the BAC+ *MyD88* heterozygous mice compared to the other two groups, although this difference was not statistically significant, presumably due to the small sample size.

## Discussion

The present study describes a severe multisystemic histiocytic inflammatory phenotype present in humanized *TLR7/8* mice that was ameliorated when the *MyD88* gene was knocked out. Activation of B cells via TLR7 signaling has been implicated in the pathogenesis of autoimmune disease such as SLE, and *Tlr7* deficient MRL/MpJ-Fas*^lpr^* (MRL/*lpr*) mice do not produce anti-Smith (sm) antigen antibodies and show lessened immune activation and clinical disease [15], [27], [28]. In previous studies of a murine lupus model, the selective deletion of *MyD88* from B cells in MRL/*lpr* mice [29], dendritic cells in *Lyn^flox/flox^Cd11c*-cre+ mice [30], and complete deletion of the *MyD88* gene in MRL/*lpr* mice [31] also prevented the generation of ANA antibodies and resolved the glomerulonephritis phenotype, but not the dermatitis phenotype. Other models of autoimmune disease, such as traditional induction of autoimmune encephalomyelitis by administration of MOG_35–55_ peptide, have been shown to be dependent on *MyD88* gene function as well [32], [33], [34]. However, in a spontaneous mouse model of experimental autoimmune encephalomyelitis, development of disease was not prevented by loss of the *MyD88* gene [35].

In the MRL/*lpr* and lupus prone *Yaa* mice, significant lesions are seen in the kidneys as a result of immune complex deposition [36]. Lesions in the kidney in MRL/*lpr* mice include inflammatory cell infiltration of the interstitium and glomerulus, mesangial proliferation, and in severe cases glomerulosclerosis and crescent formation [36]. Peribronchial and salivary gland leukocyte (predominantly histiocytic) infiltration and marked splenomegaly and lymphadenopathy are also seen in these mice [36]. The glomerular, lung, and salivary gland lesions significantly improved in *MyD88* ^−/−^ MRL/*lpr* mice [36]. The mice described here showed only mild membranoproliferative glomerulonephritis (in some BAC#2 mice), and 3 of 4 BAC#1 mice had mild to severe interstitial nephritis.

It has been shown that RNA containing antigen-antibody complexes interact with TLRs 7 and 8 to activate B lymphocytes and dendritic cells [36], [37], [38]. Blepharitis in mice with experimental SLE induced by intradermal administration of the Ig fraction of human monoclonal anti-DNA Ab 16/6Id in CFA has been described and develops approximately four to five months after administration of a booster [39]. Circulating autoantibodies, leukopenia, and proteinuria were also described in these mice [39]. To our knowledge, blepharitis has not been reported in other SLE mouse or hu*TLR8* transgenic models, although rash and dermatitis on the dorsum of the neck and back occurs in the MRL/*lpr* lupus-prone strains [15], [39], [29]. Similarly, while neuropsychiatric manifestations are relatively common in humans and in murine models of SLE, neurological symptoms in those cases are thought to be related to circulating microparticles which result in stroke, cognitive dysfunction or altered mental status, and/or seizures, as opposed to the profound meningeal infiltrate noted in the mice in this report [40].

In lupus prone mice that develop a predominantly histiocytic inflammatory multi-organ infiltrate, the origin of the histiocytes is presumed to be resident macrophages and monocytes migrating from the blood [4]. In regions that have low numbers of resident macrophages, such as the meninges, histiocytes contributing to meningitis are presumed to originate from the bone marrow and periphery via the circulation [41], [42]. A previous study has shown enhanced neuroinflammation following TLR9 agonist administration and suggested a role of normal TLR7, and potentially TLR 8 signaling, in modulating TLR9 related meningitis and neuroinflammation [41]. Given that TLR ligands activate dendritic cells and based on the histologic evidence of extramedullary hematopoeisis and increase in all cell types enumerated by FACS splenic analyses (Figure 6) in the huTLR7/8 mice, we presume that the histiocytic infiltration represented resident tissue macrophages joined by monocytes arising from the bone marrow and blood and migrating into the target organs. However, the exact origin of these histiocytes is unknown.

The mice in this report also differ from the previously reported mice with human expression of TLR8 [4]. While these humanized *TLR8* mice also developed spontaneous and severe disease of the exocrine pancreas which spared the islets, the inflammatory infiltrate was a mix of macrophages, lymphocytes, and neutrophils, and involvement of the meninges and reproductive tissues was not described [4]. Further, the human *TLR8* chimeras developed more significant disease in the kidney than the mice in the current report, and some *TLR8* chimeras developed significant clinical and histologic evidence of joint disease [4]. The mice in the current report did not develop clinical or gross evidence of joint inflammation. It has been proposed that activation of human TLR8 may cause a distinct pattern of disease than activation of TLR7, and it appears likely that activation of human TLR7 and TLR8 also results in a novel distribution and type of inflammatory infiltrate than activation of mouse TLR7 or human TLR8 alone [4].

The mice described in this report differ from the mouse models of systemic lupus erythematous in that they do not display dramatic splenomegaly or lymphadenomegaly to the degree previously described [9], [36]. Similar to mu*Tlr7* overexpression, however, all myeloid cell lineages were increased in number in hu*TLR7/8* BAC+ mice [25]. Blepharitis was consistently present in affected mice, although dermatitis was not appreciated grossly. In some mice, infiltration of the skin and subcutis of the head by histiocytic cells was noted microscopically. The mice in this report also were not leukopenic, anemic, or thrombocytopenic on complete blood count. While moderate to severe glomerular changes were seen in the BAC #1 line, only minimal to mild glomerular changes were observed in BAC #2 mice, and IgG immunohistochemistry did not show dramatically different immunopositivity between BAC #2 positive mice and control mice. Tissue from BAC #1 positive mice was not available for IgG staining. Most likely, the pathogenesis of autoimmune disease differs between traditional lupus models, in which loss of B and T cell tolerance to self antigen leads to antibody-antigen immune complex deposition in end-organs [15], [36], and the mice in this report that develop a florid multisystemic histiocytic infiltrate. Given the relatively mild renal changes in the huTLR7/8 BAC mice, antibody-antigen immune complex deposition (type III hypersensitivity reaction) is less likely to be a significant factor in disease progression. Mice of the various autoimmune prone strains (MRL/lpr, *Lyn^flox/flox^Cd11c*-cre+ mice), huTLR8 mice, and the mice of this report show phenotypic differences, and lesions can vary within autoimmune mice depending on background strain [4], [43]. This phenotypic variation may result from differential antigenic stimulation, cytokine production, and relative B or T cell contribution to the immune response, and the combination of excessive TLR7 signaling in B cells and excessive TLR8 signaling driving a distinct proinflammatory response via monocytes and myeloid dendritic cell signaling may explain why the phenotype of the huTLR7/8 mice differs from (but has components of) the previously described *Yaa* and humanized TLR8 mice [4], [15]. As has been proposed in a previous study of aging CYP1B1 knockout mice that developed glomerulonephritis and histiocytic sarcoma, defects in mononuclear cell function are present in many autoimmune prone strains of mice, and may play a role in the histiocytic proliferations noted in these huTLR7/8 mice as well [43].

It is also possible that the lesions in the huTLR7/8 BAC mice are distinct from those seen in lupus prone strains of mice with Tlr7 overexpression or in humanized TLR8 mice because they result from a neoplastic rather than an inflammatory process. Histiocytic sarcoma is an aggressive neoplasm characterized by malignant transformation of histiocytic cells of uncertain origin [44]. Histiocytic sarcoma is relatively common in older C57BL/6 mice and has a similar histologic appearance to the lesions in the hu*TLR7/8* mice, with infiltrates of round to oval large, monomorphic cells with dark central nuclei and abundant eosinophilic cytoplasm that are Mac-2 and variably F4/80 positive [44], [45], [46], [47]. Less differentiated neoplastic cells may be less likely to stain with F4/80 [48]. The presence of multinucleated giant cells also can be consistent with histiocytic sarcoma [44], [45]. Activating mutations in MyD88 have been described in human lymphomas, suggesting that constitutive human TLR7 or TLR8 signaling in our mice could be driving proliferation [49]. Further, mice from the BAC #1 lines developed earlier and more florid histiocytic proliferation more typical in appearance and showing the classic organ predilection (reproductive, hematopoietic, hepatic) of histiocytic sarcoma [44], [47], which may be related to the PRPS2 gene driving aggressive disease at an earlier age or to the site or copy number of BAC integration. However, the cell infiltrate in the mice of this report were negative on immunohistochemistry for B cell and T cell markers and were positive for macrophage markers and, to our knowledge, activating mutations in MyD88 have not been described in human or murine histiocytic sarcomas. Additionally, the lesions in the hu*TLR7/8* BAC #2 mice had less dramatic hematopoietic and hepatic involvement and had a lymphocytic component to the inflammation in the liver and kidney.

Although histiocytic sarcoma has been reported in several strains of genetically engineered mice less than 12 months of age [50], [51], [46], the neoplasm has not been reported in mice as young as the huTLR7/8 mice described in this report (in which lesions were identified grossly as early as 3 weeks of age in BAC#1 mice). Also, involvement of the lung with overtly neoplastic cells or histiocytic accumulations in previous reports of histiocytic sarcoma is present in greater than 20% of cases [50], [46], but was not observed in any of the huTLR7/8 mice. Abnormal histiocytes were not observed in the vessels of any organ on hematoxylin and eosin staining, and hyaline droplets were not observed in the renal tubular epithelial cells. Further, the severe inflammatory infiltrate in the mice of this report tended to spare structures such as the pancreatic islets. While histiocytic sarcoma is a heterogenous neoplasm that may show a range of phenotypes, tissue sites, and pattern of infiltration, especially in genetically engineered mice, previous reports suggest an older age of onset and more infiltrative tissue pattern in some cases [51], [46]. The resolution of lesions in the huTRL7/8 mice when the *MyD88* gene was knocked out may be consistent with an inflammatory etiology with inappropriate and exuberant MyD88 dependent signaling through the humanized TLR7 and TLR8 receptors. However, tumor implantation experiments would be required to more definitively characterize the infiltrate as inflammatory versus histiocytic sarcoma in these mice [46].

In conclusion, this report summarizes a novel phenotype, presumably related to replacement of the murine *Tlr7* and *Tlr8* genes with the human genes, and abnormal signaling through these TLR receptors in mice. Unfortunately, these results preclude using these mice in vaccine studies with TLR7/8 ligands.

## Supporting Information

Figure S1 **Representative lesions of the spleen, liver, lymph node, kidney and pancreas in a BAC#2 huTLR7/8 x MyD88 heterozygote 17 week old male mouse.** Lower magnification panels for architectural orientation (1.75x) paired with adjacent higher magnification images of the black boxed regions (100x). All images hematoxylin and eosin stained. A and B. Multifocal to coalescing severe (severity score 4) exocrine pancreatic histiocytic effacement with sparing of islets (one circled). C and D. Liver with mild to moderate centrilobular to bridging lymphocytic and histiocytic hepatitis. E and F. Kidney is essentially within normal limits, with only rare periglomerular histiocytic and lymphocytic inflammatory cells. G and H. Spleen with mild diffuse extramedullary hematopoeisis. I and J. Mesenteric lymph nodes with moderate sinusoidal histiocytosis and follicular germinal centers.
(JPG)

Figure S2 **Histiocytic inflammation of the kidney and liver observed in BAC#1 mice.** A. Severe histiocytic inflammation is present extending from the pole and capsule of the kidney to involve the adjacent mesenteric fat and adrenal gland. There is also a moderate multifocal to coalescing interstitial histiocytic nephritis. Hematoxylin and eosin, 20 x. B. In the liver, there is a moderate multifocal to coalescing histiocytic infiltrate affecting the periportal and, to a lesser degree, the centrilobular regions. Hematoxylin and eosin, 20x.
(TIF)
