# Supplementary figures and images for: Humanized TLR7/8 Expression Drives Proliferative Multisystemic Histiocytosis in C57BL/6 Mice
Source: PLoS One. 2014 Sep 17;9(9):e107257. doi: 10.1371/journal.pone.0107257 (PMC4168129; doi:10.1371/journal.pone.0107257)

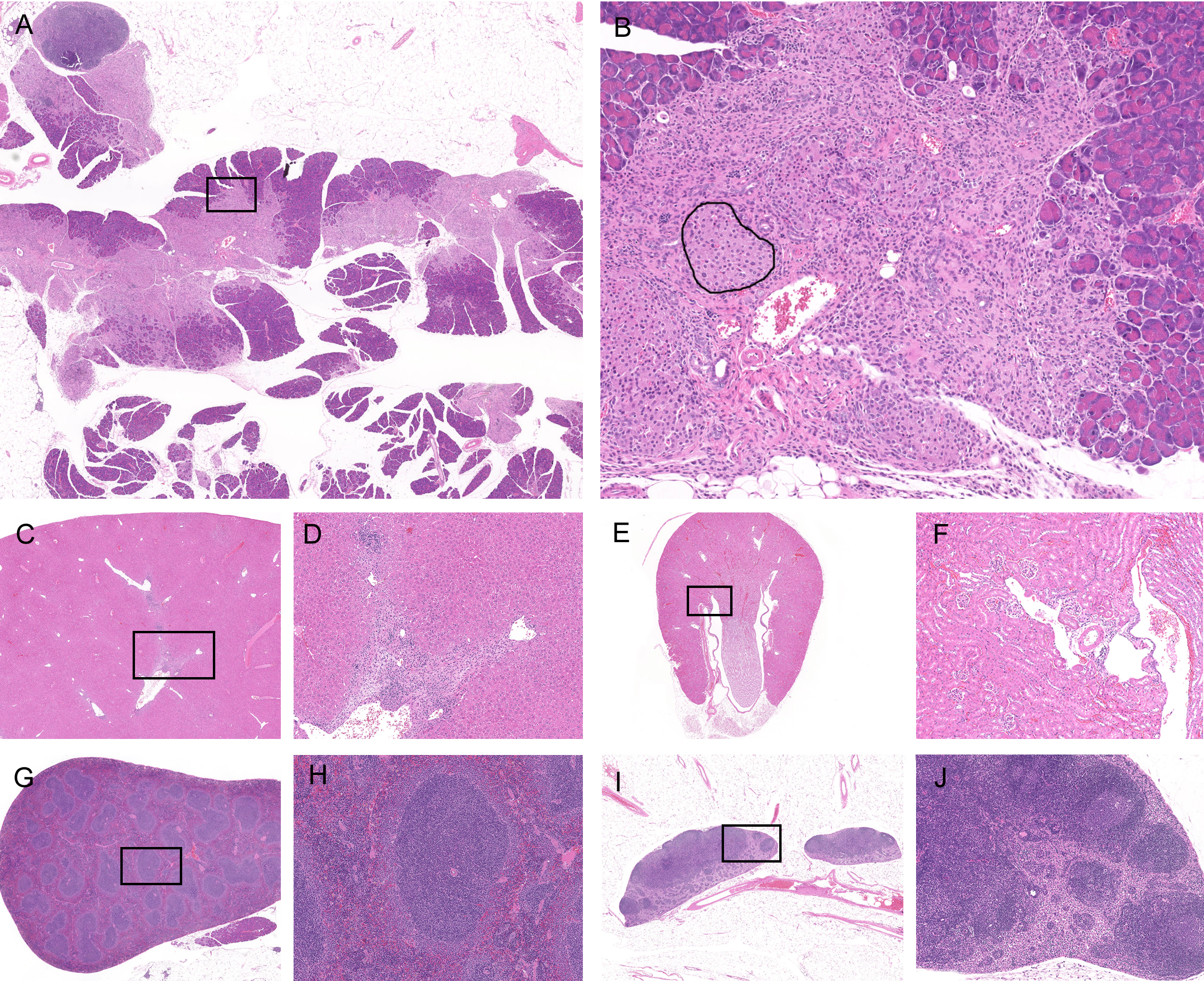

Supplement: Figure S1 — Representative lesions of the spleen, liver, lymph node, kidney and pancreas in a BAC#2 huTLR7/8 x MyD88 heterozygote 17 week old male mouse. Lower magnification panels for architectural orientation (1.75x) paired with adjacent higher magnification images of the black boxed regions (100x). All images hematoxylin and eosin stained. A and B. Multifocal to coalescing severe (severity score 4) exocrine pancreatic histiocytic effacement with sparing of islets (one circled). C and D. Liver with mild to moderate centrilobular to bridging lymphocytic and histiocytic hepatitis. E and F. Kidney is essentially within normal limits, with only rare periglomerular histiocytic and lymphocytic inflammatory cells. G and H. Spleen with mild diffuse extramedullary hematopoeisis. I and J. Mesenteric lymph nodes with moderate sinusoidal histiocytosis and follicular germinal centers. (JPG) [file pone.0107257.s001.jpg]

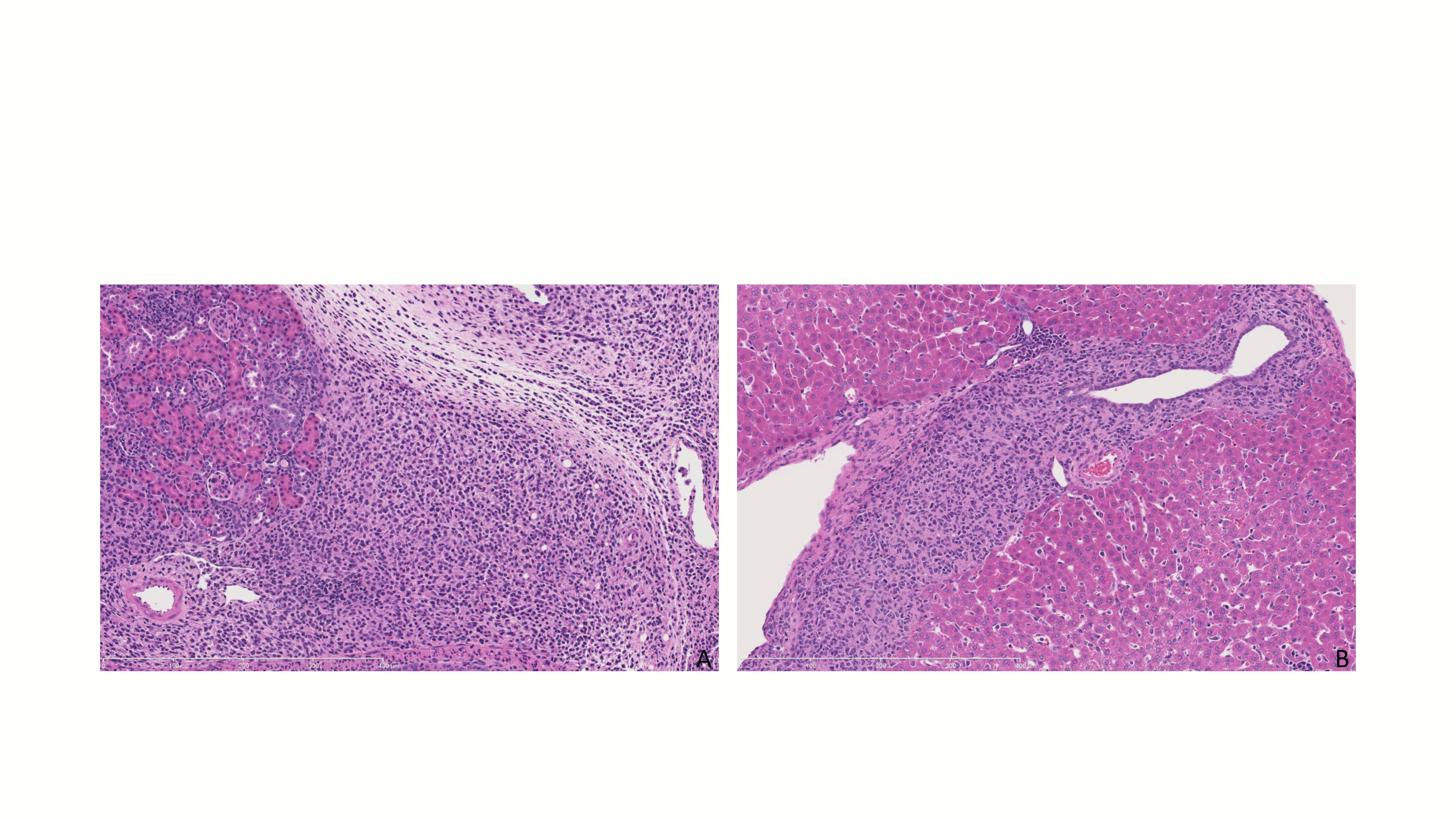

Supplement: Figure S2 — Histiocytic inflammation of the kidney and liver observed in BAC#1 mice. A. Severe histiocytic inflammation is present extending from the pole and capsule of the kidney to involve the adjacent mesenteric fat and adrenal gland. There is also a moderate multifocal to coalescing interstitial histiocytic nephritis. Hematoxylin and eosin, 20 x. B. In the liver, there is a moderate multifocal to coalescing histiocytic infiltrate affecting the periportal and, to a lesser degree, the centrilobular regions. Hematoxylin and eosin, 20x. (TIF) [file pone.0107257.s002.tif]
